# Risk factors and predictive models for early death in patients with advanced melanoma: A population-based study

**DOI:** 10.1097/MD.0000000000035380

**Published:** 2023-10-06

**Authors:** Siru Li, Cunli Yin, Xi Yang, Yingchun Lu, ChunYu Wang, Bin Liu

**Affiliations:** a School of Medicine, University of Electronic Science and Technology of China, Chengdu, China; b School of Medical and Life Sciences, Chengdu University of Traditional Chinese Medicine, Chengdu, China; c Department of Medical Oncology, Sichuan Clinical Research Center for Cancer, Sichuan Cancer Hospital and Institute, Sichuan Cancer Center, Affiliated Cancer Hospital of University of Electronic Science and Technology of China, Chengdu, China.

**Keywords:** advanced melanoma, early death, Epidemiology and End Results (SEER), nomogram, predictive model, surveillance

## Abstract

The prognosis for advanced melanoma (AM) is extremely poor. Some patients are already in an advanced stage at the time of their first diagnosis and face a significant risk of early death. This study predicted all-cause early death and cancer-specific early death in patients with AM by identifying independent risk factors, building 2 separate nomogram models, and validating the efficiency of the models. A total of 2138 patients diagnosed with AM from 2010 to 2015 were registered in the Surveillance, Epidemiology and End Results (SEER) database and randomly assigned to a training cohort and a validation cohort. Logistic regression models were used to identify the associated independent risk factors. These factors have also been used to build nomograms for early deaths. Next, we validated the model’s predictive power by examining subject operating characteristic curves, then applied calibration curves to assess the accuracy of the models, and finally, tested the net benefit of interventions based on decision curve analysis. The results of the logistic regression model showed that marital status, primary site, histological type, N stage, surgery, chemotherapy, bone, liver, lung and brain metastases were significant independent risk factors for early death. These identified factors contributed to the creation of 2 nomograms, which predict the risk of all-cause early death and cancer-specific early death in patients with AM. In the all-cause early death model, the area under the curve was 0.751 and 0.759 for the training and validation groups, respectively, whereas in the cancer-specific early death model, the area under the curve was 0.740 and 0.757 for the training and validation groups. Calibration curves indicated a high degree of agreement between the predicted and observed probabilities, and the decision curve analysis demonstrated a high value for the model in terms of its applicability in clinical settings. These nomograms have practical applications in predicting the risk of early death in patients with AM, helping oncologists to intervene early and develop more personalized treatment strategies.

## 1. Introduction

Malignant melanoma is one of the most important and most severe types of skin tumors and is extremely aggressive.^[1]^ Its incidence has increased rapidly over the past 50 years.^[2,3]^ It tends to metastasize early in the progression of the disease. Many patients have metastasized at the time of diagnosis or experienced recurrence after initial curative treatment.^[4,5]^ The most common sites of metastases from melanoma are the skin and subcutaneous tissue, followed by distant metastases in the lung, liver, bone and brain.^[6,7]^ Despite tremendous advances in the treatment of advanced malignant melanoma with molecularly targeted therapies and immunotherapy,^[8,9]^ the prognosis for advanced metastatic melanoma remains extremely poor, with a 5-year survival rate of less than 10% and a median overall survival (OS) of approximately 7.5 months.^[5]^

Clinicians currently assess the prognosis of melanoma primarily based on the American Joint Committee on Cancer (AJCC) 8th edition TNM staging system.^[7]^ However, it has been well observed that despite the international staging system has been adopted, the variation in the conclusions reached by pathologists in the diagnosis of malignant melanoma is extremely high between different observers. As a result, the diagnosis of melanoma remains difficult to be accurate and consistent.^[7,9]^ Therefore, the prognosis of melanoma is difficult to predict accurately by relying on the TNM staging alone.

Nomograms have been widely used in the medical field to predict the survival rate of tumor patients. Compared with the traditional TNM staging system, it can incorporate more possible prognostic factors and is more accurate in prognostic prediction.

Based on some previous studies,^[10–12]^ we defined early death as death within 3 months of the first diagnosis. Therefore, in this study, we extracted and analyzed data from the Surveillance, Epidemiology, and End Results (SEER) database for patients diagnosed with advanced melanoma (AM) between 2010 and 2015. Risk factors associated with early death in AM patients were identified and developed nomograms to assess the prognosis of these patients more accurately. It helps oncologists to identify and intervene early and provide more individualized treatment plans.

## 2. Method

### 2.1. Patients selection

We conducted a retrospective cohort study using data from the SEER database, including demographic, clinicopathological, and survival data of cancer patients. This study used SEER*Stat software (www.seer.cancer.gov, software version 8.4.0) to extract data from the SEER database for patients diagnosed with AM from 2010 to 2015, applying the International Classification of Diseases for Oncology, and 3rd edition (ICD-O-3) criteria, to identify patients with melanoma based on the primary site (ICD-10/C43-C44), whose diagnosis was also confirmed histologically.

**Figure 1.**
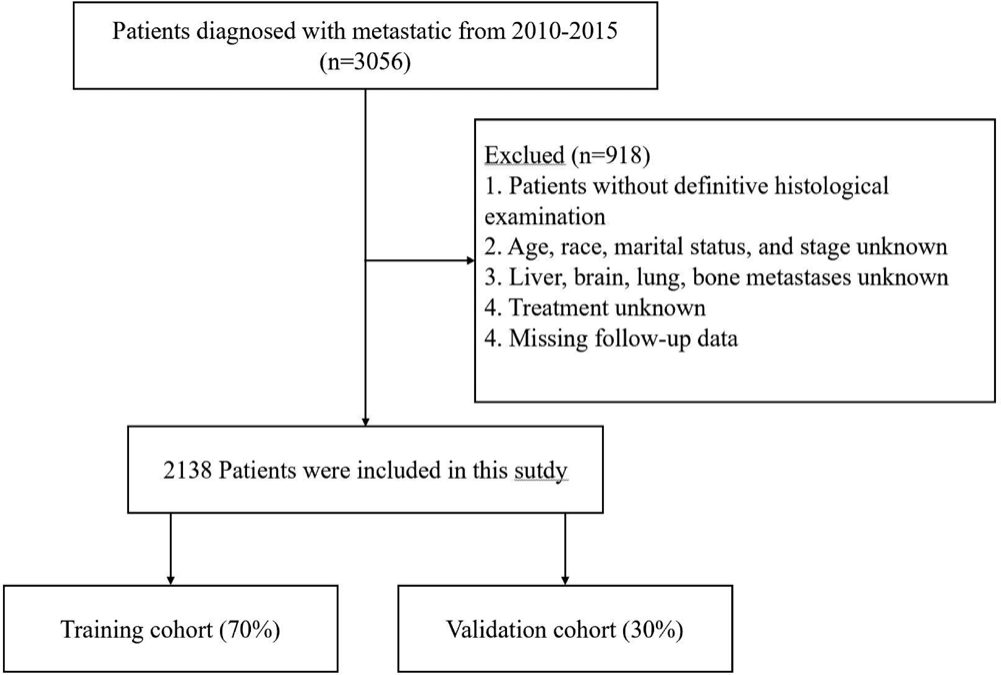
Flow chart of patient selection for included studies.

The exclusion criteria were as follows: patients without definitive histological examination; Unknown age, race, marital status, and stage; Liver, brain, lung, bone metastases unknown; Treatment unknown; and Missing follow-up data. The specific patient selection flow chart is shown in Figure 1. Based on the inclusion and exclusion criteria, a total of 2138 patients were ultimately included in the study and randomly assigned by computer to the training group cohort and the validation cohort in a 7:3 ratio.

### 2.2. Statistical analysis

The endpoint of this study was the survival time, and patients who died within 3 months after the initial diagnosis were included in this study. All included variables except the survival time are described as numbers and percentages (N, %). As described above, these patients were randomly assigned to the training and validation cohorts. The training cohort was used to identify prognostic factors associated with early death in patients with AM and to develop nomograms. The nomogram is a common tool for prognostic evaluation of tumors, which transforms complex regression equations into visual graphs, making the results of predictive models more readable and facilitating patient assessment. With Nomogram, developers can provide an accurate digital probability of survival or risk for each patient, which can assist physicians in decision making and develop more personalized treatment strategies.

Variables that were significantly associated with early death in the multifactorial logistic analysis were included in the nomogram, and we developed nomograms for cancer-specific early death and all-cause early death, respectively. The validation cohort was used to verify the accuracy and efficiency of the model. Pearson chi-square tests were used to explore the differences between this training and validation cohorts.

Statistical analysis of the data was performed using R software (version 4.2.2) and SPSS statistics software (version 27, IBM Corp, Armonk, NY), and a two-sided *P* value < .05 was considered statistically significant.

After the model was built, to assess the reliability of the nomogram, we calculated the area under the curve (AUC) by ROC curves in both the training and validation cohorts to predict model efficacy, applied calibration curves to assess the model’s agreement with reality, and finally, tested the net benefit of interventions based on model results by decision curve analysis, which assesses the value of clinical application. The calibration curves represent the agreement between observed and predicted probabilities through 1000 resampling bootstraps.

## 3. Result

### 3.1. Demographic and clinical features of AM

Based on the inclusion and exclusion criteria, 2138 patients diagnosed with AM from 2010 to 2015 in the SEER database were included in this study (Fig. 1), and were randomized in a ratio of 7:3 into a training cohort and a validation cohort. Demographic and clinicopathological characteristics of patients in the training cohort (n = 1498) and validation cohort (n = 640) are presented in Table 1. Overall, there was a predominance of patients over 40 years old, and 70.2% were male patients (n = 1500). More than 90% of the patients (n = 2060) were white. Among the primary sites, 63.0% of the patients had melanoma of unknown origin (n = 1348), 32.9% had cutaneous melanoma (n = 703), and a very small percentage had primary sites in the external ear or choroid and other rare sites. The majority of patients had the pathologic type of malignant melanoma, which accounted for 83.3% of the total (n = 1781), nodular melanoma accounted for 8.9% (n = 191), and the rest of the pathologic types were less common. Most of these patients (73.2%) did not undergo surgery (n = 1564), while 42.6% of them received radiotherapy and 30.6% received chemotherapy. The percentage of patients with metastatic lesions of the liver, lungs, bone, and brain were: 29.7%, 50.7%, 25.1%, and 41.1%, respectively.

**Table 1:** Baseline features for training and validation cohorts.

Characteristics	Number of patients (%)			*P* value
	Overall (n = 2138)	Training cohort (n = 1498)	Validation cohort (n = 640)	
Age				
** **<40	135 (6.3)	90 (6.0)	45 (7.0)	.619
** **40–65	1024 (47.9)	724 (48.3)	300 (46.9)	
** **>65	979 (45.8)	684 (45.7)	295 (46.1)	
Sex				
Male	1500 (70.2)	1042 (69.6)	458 (71.6)	.381
Female	638 (29.8)	456 (30.4)	182 (28.4)	
Race				
White	2060 (96.4)	1439 (96.1)	621 (97.0)	.516
Black	30 (1.4)	22 (1.5)	8 (1.2)	
Other	48 (2.2)	37 (2.5)	11 (1.7)	
Marital				
Married	1157 (54.1)	796 (53.1)	361 (56.4)	.18
Unmarried	981 (45.9)	702 (46.9)	279 (43.6)	
Primary site				
Skin, NOS	1348 (63.0)	957 (63.9)	391 (61.1)	.795
Skin other/unspecific parts of face	48 (2.2)	32 (2.1)	16 (2.5)	
Skin of scalp and neck	121 (5.7)	88 (5.9)	33 (5.2)	
Skin of trunk	272 (12.7)	183 (12.2)	89 (13.9)	
Skin of upper limb and shoulder	113 (5.3)	82 (5.5)	31 (4.8)	
Skin of lower limb and hip	149 (7.0)	97 (6.5)	52 (8.1)	
Choroid	23 (1.1)	16 (1.1)	7 (1.1)	
External ear	17 (0.8)	11 (0.7)	6 (0.9)	
Other rare sites	47 (2.2)	32 (2.1)	15 (2.3)	
Melanoma type				
Malignant melanoma, NOS	1781 (83.3)	1256 (83.8)	525 (82.0)	.192
Nodular melanoma	191 (8.9)	126 (8.4)	65 (10.2)	
Superficial spreading melanoma	40 (1.9)	29 (1.9)	11 (1.7)	
Spindle cell melanoma, NOS	36 (1.7)	20 (1.3)	16 (2.5)	
Amelanotic melanoma	28 (1.3)	23 (1.5)	5 (0.8)	
Other rare types	62 (2.9)	44 (2.9)	18 (2.8)	
T Stage				
T0	939 (43.9)	661 (44.1)	278 (43.4)	.451
T1–T2	164 (7.7)	123 (8.2)	41 (6.4)	
T3–T4	389 (18.2)	266 (17.8)	123 (19.2)	
TX	646 (30.2)	448 (29.9)	198 (30.9)	
N Stage				
N0	828 (38.7)	572 (38.2)	256 (40.0)	.143
N1	416 (19.5)	276 (18.4)	140 (21.9)	
N2	86 (4.0)	63 (4.2)	23 (3.6)	
N3	145 (6.8)	101 (6.7)	44 (6.9)	
NX	663 (31.0)	486 (32.4)	177 (27.7)	
Surgery				
No	1564 (73.2)	1110 (74.1)	454 (70.9)	.145
Yes	574 (26.8)	388 (25.9)	186 (29.1)	
Radiation				
No	1227 (57.4)	878 (58.6)	349 (54.5)	.089
Yes	911 (42.6)	620 (41.4)	291 (45.5)	
Chemotherapy				
No	1484 (69.4)	1050 (70.1)	434 (67.8)	.319
Yes	654 (30.6)	448 (29.9)	206 (32.2)	
Bone metastasis				
No	1602 (74.9)	1133 (75.6)	469 (73.3)	.273
Yes	536 (25.1)	365 (24.4)	171 (26.7)	
Brain metastasis				
No	1260 (58.9)	872 (58.2)	388 (60.6)	.322
Yes	878 (41.1)	626 (41.8)	252 (39.4)	
Liver metastasis				
No	1504 (70.3)	1051 (70.2)	453 (70.8)	.813
Yes	634 (29.7)	447 (29.8)	187 (29.2)	
Lung metastasis				
** **No	1053 (49.3)	724 (48.3)	329 (51.4)	.209
** **Yes	1085 (50.7)	774 (51.7)	311 (48.6)	

### 3.2. Mortality of early death

Among the 2138 patients with AM, 811 (37.9%) died early. Out of these, 767 patients died from cancer-specific causes, while 44 patients died from non-cancer-specific causes. Early mortality increased progressively with age and was much higher in men than in women. The vast majority of patients who died early did not receive surgery (n = 691, 85.2%) or chemotherapy (n = 668, 82.4%), and the study also showed that patients with lung and brain metastases were more likely to die early. Table 2 provides a detailed description of the demographic and clinicopathologic characteristics of patients with AM, distinguishing between those who experienced early death and those who did not.

**Table 2:** Baseline characteristics of all-cause early death and cancer-specific early death in patients with advanced melanoma.

Characteristics	Number of patients (%)			Cancer-specific early death (n = 767)
	Overall (n = 2138)	No early death (n = 1327)	All-cause early death (n = 811)	
Age				
** **<40	135 (6.3)	94 (7.1)	41 (5.1)	40 (5.2)
** **40–65	1024 (47.9)	675 (50.9)	349 (43.0)	336 (43.8)
** **>65	979 (45.8)	558 (42.0)	421 (51.9)	391 (51.0)
Sex				
Male	1500 (70.2)	923 (69.6)	577 (71.1)	546 (71.2)
Female	638 (29.8)	404 30.4)	234 (28.9)	221 (28.8)
Race				
White	2060 (96.4)	1274 (96.0)	786 (96.9)	743 (96.9)
Black	30 (1.4)	20 (1.5)	10 (1.2)	10 (1.3)
Other	48 (2.2)	33 (2.5)	15 (1.8)	14 (1.8)
Marital				
Married	1157 (54.1)	764 (57.6)	393 (48.5)	373 (48.6)
Unmarried	981 (45.9)	563 (42.4)	418 (51.5)	394 (51.4)
Primary site				
Skin, NOS	1348 (63.0)	744 (56.1)	604 (74.5)	572 (74.6)
Skin other/unspecific parts of face	48 (2.2)	35 (2.6)	13 (1.6)	12 (1.6)
Skin of scalp and neck	121 (5.7)	97 (7.3)	24 (3.0)	24 (3.1)
Skin of trunk	272 (12.7)	186 (14.0)	86 (10.6)	77 (10.0)
Skin of upper limb and shoulder	113 (5.3)	76 (5.7)	37 (4.6)	35 (4.6)
Skin of lower limb and hip	149 (7.0)	118 (8.9)	31 (3.8)	31 (4.0)
Choroid	23 (1.1)	20 (1.5)	3 (0.4)	3 (0.4)
External ear	17 (0.8)	14 (1.1)	3 (0.4)	3 (0.4)
Other rare sites	47 (2.2)	37 (2.8)	10 (1.2)	10 (1.3)
Melanoma type				
Malignant melanoma, NOS	1781 (83.3)	1055 (79.5)	726 (89.5)	684 (89.2)
Nodular melanoma	191 (8.9)	150 (11.3)	41 (5.1)	41 (5.3)
Superficial spreading melanoma	40 (1.9)	32 (2.4)	8 (1.0)	7 (0.9)
Spindle cell melanoma, NOS	36 (1.7)	25 (1.9)	11 (1.4)	11 (1.4)
Amelanotic melanoma	28 (1.3)	14 (1.1)	14 (1.7)	13 (1.7)
Other rare types	62 (2.9)	51 (3.8)	11 (1.4)	11 (1.4)
T Stage				
T0	939 (43.9)	547 (41.2)	392 (48.3)	373 (48.6)
T1–T2	164 (7.7)	127 (9.6)	37 (4.6)	36 (4.7)
T3–T4	389 (18.2)	292 (22.0)	97 (12.0)	93 (12.1)
TX	646 (30.2)	361 (27.2)	285 (35.1)	265 (34.6)
N Stage				
N0	828 (38.7)	538 (40.5)	290 (35.8)	273 (35.6)
N1	416 (19.5)	282 (21.3)	144 (17.8)	140 (18.3)
N2	86 (4.0)	72 (5.4)	14 (1.7)	14 (1.8)
N3	145 (6.8)	108 (8.1)	37 (4.6)	32 (4.2)
NX	663 (31.0)	337 (25.4)	326 (40.2)	308 (40.2)
Surgery				
No	1564 (73.2)	873 (65.8)	691 (85.2)	651 (84.9)
Yes	574 (26.8)	454 (34.2)	120 (14.8)	116 (15.1)
Radiation				
No	1227 (57.4)	738 (55.6)	489 (60.3)	455 (59.3)
Yes	911 (42.6)	589 (44.4)	322 (39.7)	312 (40.7)
Chemotherapy				
No	1484 (69.4)	816 (61.5)	668 (82.4)	628 (81.9)
Yes	654 (30.6)	511 (38.5)	143 (17.6)	139 (18.1)
Bone metastasis				
No	1602 (74.9)	1032 (77.8)	570 (70.3)	542 (70.7)
Yes	536 (25.1)	295 (22.2)	241 (29.7)	225 (29.3)
Brain metastasis				
No	1260 (58.9)	862 (65.0)	398 (49.1)	373 (48.6)
Yes	878 (41.1)	465 (35.0)	413 (50.9)	394 (51.4)
Liver metastasis				
No	1504 (70.3)	1017 (76.6)	487 (60.0)	455 (59.3)
Yes	634 (29.7)	310 (23.4)	324 (40.0)	312 (40.7)
Lung metastasis				
** **No	1053 (49.3)	718 (54.1)	335 (41.3)	314 (40.9)
** **Yes	1085 (50.7)	609 (45.9)	476 (58.7)	453 (59.1)

### 3.3. Identification of early death prognostic factor

Risk factors associated with early death in the training cohort were first analyzed by univariate logistic regression. The results showed that age, marital status, primary site, type of pathology, T stage, N stage, surgery, chemotherapy, bone metastasis, lung metastasis, liver metastasis, brain metastasis, and marital status were all significant risk factors for all-cause early death. However, among them, age was not a risk factor for cancer-specific early death. A more detailed presentation of the results of the univariate analysis can be seen in Table 3. Significant risk factors identified in the univariate logistic regression analysis were included in the multivariate logistic analysis. The multivariate analysis revealed that all of the above significant risk factors, except age and T-stage, were independent risk factors for predicting all-cause early death and cancer-specific early death in patients with AM. More detailed information can be seen in Table 4.

**Table 3:** Univariate logistic regression analysis of the training cohort.

Variable	All-cause early death			Cancer-specific early death		
	OR	95% CI	*P* value	OR	95% CI	*P* value
Age						
<40	1.1	0.69–1.75	.701	1.08	0.67–1.73	.757
40–65	1.7	1.07–2.72	.026	1.55	0.97–2.49	.067
>65	1 (ref)			1 (ref)		
Sex						
Male	1 (ref)			1 (ref)		
Female	0.95	0.76–1.2	.69	0.97	0.77–1.22	.77
Race						
White	1 (ref)			1 (ref)		
Black	1.08	0.46–2.54	.86	1.2	0.51–2.82	.679
Other	0.75	0.37–1.5	.416	0.73	0.36–1.49	.391
Marital						
Married	1 (ref)			1 (ref)		
Unmarried	1.4	1.14–1.72	.002	1.35	1.09–1.66	.006
Primary site						
Skin, NOS	1 (ref)			1 (ref)		
Skin other/unspecific parts of face	0.54	0.25–1.16	.115	0.52	0.24–1.14	.103
Skin of scalp and neck	0.27	0.15–0.46	<.001	0.3	0.17–0.52	<.001
Skin of trunk	0.64	0.46–0.89	.009	0.6	0.43–0.85	.003
Skin of upper limb and shoulder	0.59	0.36–0.95	.029	0.59	0.36–0.95	.031
Skin of lower limb and hip	0.31	0.19–0.52	<.001	0.35	0.21–0.58	<.001
Choroid	0.08	0.01–0.61	.014	0.09	0.01–0.68	.019
External ear	0.27	0.06–1.24	.091	0.3	0.06–1.38	.121
Other rare sites	0.33	0.14–0.78	.011	0.37	0.16–0.87	.023
Melanoma type						
Malignant melanoma, NOS	1 (ref)			1 (ref)		
Nodular melanoma	0.46	0.3–0.7	<.001	0.51	0.34–0.78	.002
Superficial spreading melanoma	0.45	0.19–1.06	.066	0.41	0.17–1.02	.054
Spindle cell melanoma, NOS	0.47	0.17–1.3	.145	0.52	0.19–1.45	.214
Amelanotic melanoma	1.08	0.47–2.49	.853	1.01	0.43–2.36	.978
Other rare types	0.31	0.14–0.68	.003	0.35	0.16–0.76	.008
T Stage						
T0	1 (ref)			1 (ref)		
T1–T2	0.34	0.22–0.55	<.001	0.36	0.22–0.57	<.001
T3–T4	0.51	0.38–0.7	<.001	0.52	0.38–0.71	<.001
TX	1.13	0.89–1.44	.319	1.06	0.83–1.35	.654
N Stage						
N0	1 (ref)			1 (ref)		
N1	1.06	0.78–1.43	.718	1.09	0.8–1.47	.58
N2	0.27	0.13–0.58	.001	0.3	0.14–0.64	.002
N3	0.68	0.42–1.09	.108	0.6	0.37–0.99	.045
NX	1.94	1.51–2.48	<.001	1.87	1.45–2.39	<.001
Surgery						
No	1 (ref)			1 (ref)		
Yes	0.34	0.26–0.44	<.001	0.36	0.28–0.48	<.001
Radiation						
No	1 (ref)			1 (ref)		
Yes	0.82	0.66–1.01	.063	0.88	0.71–1.09	.257
Chemotherapy						
No	1 (ref)			1 (ref)		
Yes	0.32	0.25–0.41	<.001	0.35	0.27–0.45	<.001
Bone metastasis						
No	1 (ref)			1 (ref)		
Yes	1.48	1.16–1.87	.001	1.42	1.11–1.8	.005
Brain metastasis						
No	1 (ref)			1 (ref)		
Yes	1.89	1.53–2.34	<.001	1.9	1.53–2.35	<.001
Liver metastasis						
No	1 (ref)			1 (ref)		
Yes	1.94	1.55–2.42	<.001	1.98	1.58–2.48	<.001
Lung metastasis						
No	1 (ref)			1 (ref)		
Yes	1.76	1.43–2.17	<.001	1.74	1.4–2.15	<.001

**Table 4:** Multivariate logistic regression analysis of the training cohort.

Variable	All-cause early death			Cancer-specific early death		
	OR	95% CI	*P* value	OR	95% CI	*P* value
Age						
<40	1 (ref)			NA		
40–65	0.88	0.51–1.5	.636			
>65	1.41	0.82–2.44	.212			
Marital						
Married	1 (ref)			1 (ref)		
Unmarried	1.48	1.16–1.88	.002	1.37	1.08–1.73	.01
Primary site						
Skin, NOS	1 (ref)			1 (ref)		
Skin other/unspecific parts of face	1.42	0.56–3.59	.461	1.23	0.48–3.13	.665
Skin of scalp and neck	0.47	0.23–0.97	.04	0.53	0.26–1.08	.078
Skin of trunk	1.16	0.68–1.98	.579	0.98	0.58–1.66	.938
Skin of upper limb and shoulder	1.24	0.64–2.38	.522	1.15	0.6–2.21	.667
Skin of lower limb and hip	0.6	0.31–1.18	.14	0.66	0.34–1.28	.219
Choroid	0.09	0.01–0.76	.027	0.1	0.01–0.82	.032
External ear	0.59	0.1–3.58	.567	0.67	0.12–3.83	.648
Other rare sites	0.42	0.15–1.13	.086	0.5	0.19–1.34	.168
Melanoma type						
Malignant melanoma, NOS	1 (ref)			1 (ref)		
Nodular melanoma	0.91	0.51–1.62	.757	0.97	0.55–1.71	.911
Superficial spreading melanoma	0.76	0.28–2.09	.594	0.67	0.24–1.88	.448
Spindle cell melanoma, NOS	0.54	0.17–1.68	.286	0.66	0.22–2.01	.468
Amelanotic melanoma	1.09	0.44–2.66	.857	1.02	0.41–2.54	.962
Other rare types	0.54	0.23–1.3	.171	0.64	0.27–1.51	.31
T stage						
T0	1 (ref)			1 (ref)		
T1–T2	1.08	0.53–2.22	.824	1.09	0.53–2.21	.82
T3–T4	1.46	0.78–2.71	.236	1.44	0.78–2.66	.249
TX	1.22	0.91–1.63	.19	1.16	0.87–1.55	.317
N Stage						
N0	1 (ref)			1 (ref)		
N1	1.29	0.91–1.82	.147	1.27	0.9–1.79	.168
N2	0.39	0.17–0.9	.027	0.4	0.18–0.91	.03
N3	1.16	0.66–2.04	.613	0.95	0.54–1.7	.874
NX	1.77	1.33–2.35	0	1.67	1.26–2.21	0
Surgery						
No	1 (ref)			1 (ref)		
Yes	0.42	0.27–0.66	0	0.48	0.31–0.75	.001
Chemotherapy						
No	1 (ref)			1 (ref)		
Yes	0.27	0.2–0.36	0	0.29	0.22–0.38	0
Bone metastasis						
No	1 (ref)			1 (ref)		
Yes	1.5	1.13–2	.005	1.33	1–1.77	.047
Brain metastasis						
No	1 (ref)			1 (ref)		
Yes	2	1.56–2.55	0	1.91	1.5–2.43	0
Liver metastasis						
No	1 (ref)			1 (ref)		
Yes	2.23	1.7–2.92	0	2.21	1.69–2.88	0
Lung metastasis						
No	1 (ref)			1 (ref)		
Yes	1.45	1.14–1.84	.003	1.46	1.15–1.86	.002

### 3.4. Nomogram construction and validation

**Figure 2.**
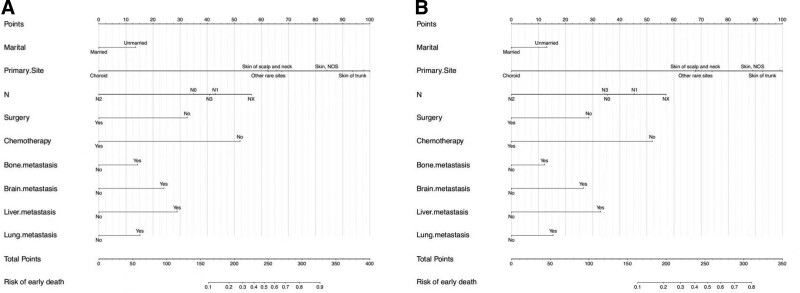
Nomograms for predicting all-cause (A) and cancer-specific early death (B) in advanced melanoma patients.

Based on the independent risk factors identified by multivariate logistic regression, we constructed 2 independent nomograms to predict the risk of all-cause early death (Fig. 2A) and cancer-specific early death (Fig. 2B) in patients with AM, respectively. Each influential factor in the model was assigned a score for each value level taken according to the degree of contribution of each factor to the outcome variable (magnitude of the regression coefficient), and then the individual scores were summed to obtain the total score. The probabilities of all-cause early death and cancer-specific early death ranged from 0.10 to 0.90, and the total score for most patients ranged from 150 to 350. From the nomogram, it was found that chemotherapy and N-stage primary site had a good prognostic value for predicting early death.

**Figure 3.**
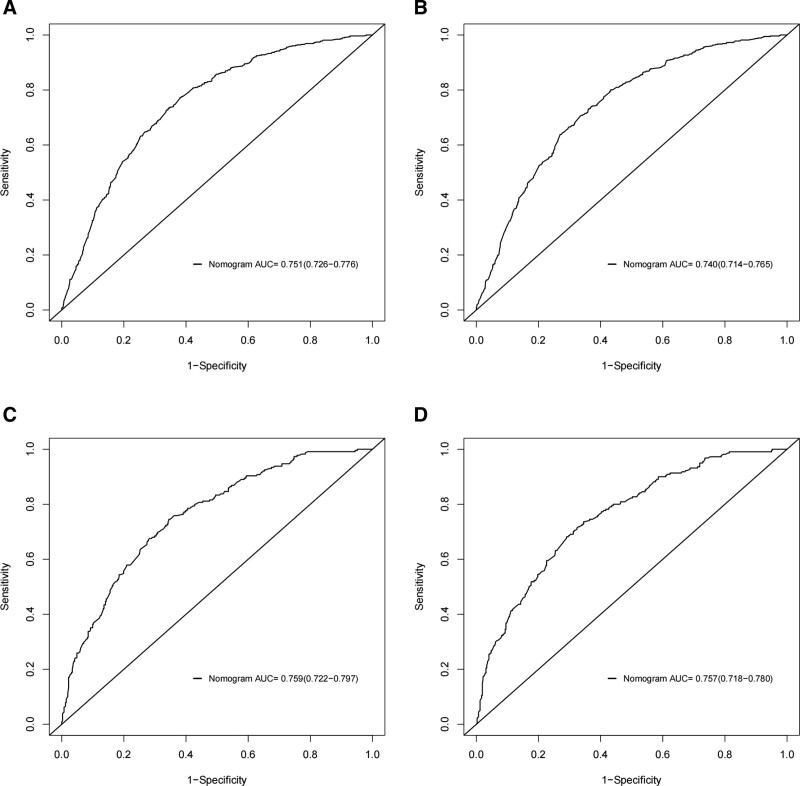
ROC for discrimination of nomograms in predicting all-cause and cancer-specific early death in the training cohort (A, B) and the validation cohort (C, D). AUC indicates area under the curve, with higher values indicating higher prediction accuracy. AUC = area under the ROC curves, ROC = operating characteristic curves.

**Figure 4.**
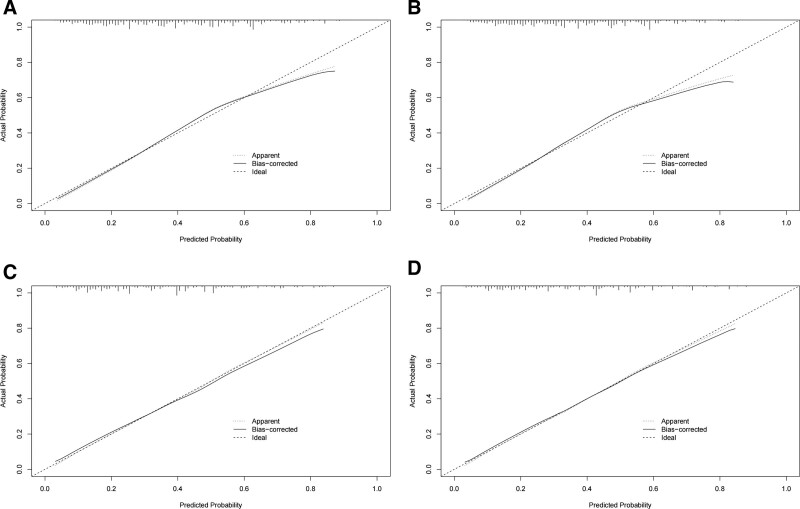
Calibration curves for assessing the calibration of the nomogram in predicting all-cause early death and cancer-specific early death in the training cohort (A, B) and the validation cohort (C, D).

**Figure 5.**
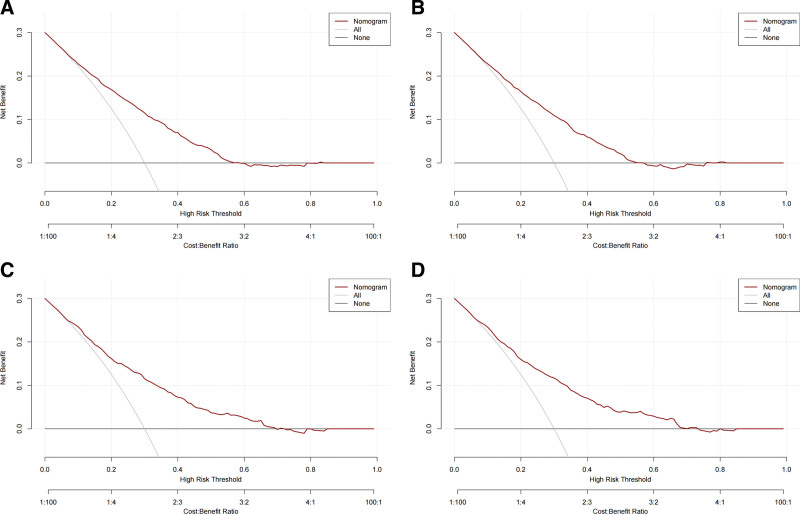
DCA for the nomograms in predicting all-cause early death and cancer-specific early death in the training cohort (A, B) and the validation cohort (C, D). DCA = decision curve analysis.

The ROC curves of the nomogram used to assess overall and cancer-specific early death are shown in Figure 3. The AUC for overall early death in the training group was 0.751 (Fig. 3A; 95% CI: 0.726–0.776), while the AUC for cancer-specific early death was 0.794 (Fig. 3B; 95% CI: 0.714–0.765). The AUC for overall early death in the validation group was 0.759 (Fig. 3C; 95% CI: 0.722–0.797) and the AUC for cancer-specific early death was 0.757 (Fig. 3D; 95% CI: 0.718–0.780), respectively. The calibration plots of the model showed that the calibration curves fit the diagonal line relatively well, indicating that the predicted early death was highly consistent with the actual outcome (Fig. 4), while the decision curve analysis showed that the model has a high value for clinical application (Fig. 5). In the training group, the sensitivity, specificity, and odds ratio for all-cause early death were 0.732, 0.685, and 5.959, respectively. For cancer-specific early death, they were 0.729, 0.669, and 5.443. While in the validation set, the sensitivity, specificity, and odds ratio for all-cause early death were 0.716, 0.655, and 5.573, respectively. For cancer-specific early death, the data were 0.695, 0.705, and 5.451.

## 4. Discussion

The prognosis for AM is extremely poor, with a 5-year survival rate of less than 10%.^[5,13]^ With the successful development of targeted and immunotherapy for patients with metastatic melanoma, there has been a significant improvement in quality of life and overall survival,^[14,15]^ but mortality rate for patients with AM remained high, with more than one-third of the patients in this study dying within 3 months of diagnosis. However, few studies have assessed early death in AM, so it is essential to explore the risk factors that lead to early death in order to increase patient survival as much as possible. To our knowledge, this is the first study to identify risk factors and construct a column chart for identifying early death in AM.

Melanoma is a highly heterogeneous disease, and despite clinical staging guidelines, the prognosis of metastatic melanoma remains variable and unpredictable. Several current studies suggest that nomograms can be used to predict the prognosis of patients with melanoma. Li et al^[16]^ developed a predictive nomogram for cancer-specific death (CSD) in metastatic melanoma patients and a corresponding risk classification system was developed, with training cohort AUC values of 0.706, 0.700, and 0.706, respectively. A study by Du et al^[17]^ combined a predictive model of the ki-67 clinical factor for predicting the prognosis of extremity melanoma.

The data in this study suggest that marital status, primary site, histological type, N stage, surgery, chemotherapy, bone, liver, lung and brain metastases are associated with the risk of all-cause early death and cancer-specific early death. The results of univariate and multivariate analyses demonstrate the significant prognostic value of the TNM staging for metastatic melanoma. A study conducted by Cozzolino et al^[18]^ showed that tumor site, tissue type, distant metastases, were significantly associated with overall survival, which is in agreement with our findings. In this study, we incorporated both TNM staging, clinical and pathological features, and identified a total of 10 independent risk factors to construct nomograms for predicting early death in patients with AM. The area under the curve (AUC) for overall early death in the training group was 0.751, while the AUC for cancer-specific early death was 0.794. In comparison, a previous study on the prognostic accuracy of the AJCC staging system, version 8, with a total cohort of 1462 patients, reported an AUC of 0.74.^[19]^

In patients with advanced disease, aggressive treatment is an important means of reducing early mortality. Both surgery and chemotherapy can reduce the risk of early death. Appropriate surgical treatment is essential for the diagnosis, staging, and treatment of melanoma. First, biopsy surgery provides clarity in the diagnosis and staging of melanoma and also helps prevent surgical site errors.^[20]^ Second, enlarged excision of the primary melanoma site can reduce the risk of local recurrence.^[21,22]^ In patients with a single metastasis or a few metastases, surgical excision of all metastases can occasionally lead to lasting improvement.^[23,24]^ However, in patients with mucosal melanoma, local recurrence is common even with aggressive surgical treatment, and most patients die from distant lesions rather than from uncontrolled local lesions.^[25,26]^ Overall, deciding whether to operate on a patient with metastatic melanoma is a complex and often difficult process, regardless of whether the aim is curative or palliative treatment.

Metastases lead to a significant risk of early death, with over 95% of early deaths being metastatic melanoma and leading to cancer-specific death. Lung is the most common site of distant metastases from melanoma, accounting for as much as 40% of stage IV disease, and lung metastasectomy may improve survival,^[27]^ In today’s era of targeted and immune checkpoint blockade therapy, the choice of lung metastasectomy should also take into account the patient’s outcome with systemic therapy.^[28–30]^ Brain metastases are a common complication for patients with regionally advanced and metastatic melanoma, and a significant cause of complications and death. Various tools have been developed to determine the prognosis of patients with melanoma brain metastases.^[31]^ Once patients at a high risk of premature death are identified, oncologists may be able to give more advanced treatment strategies, such as clinical trials, and closer follow-up.

The present study also has some unavoidable limitations, such as the model did not incorporate some known risk factors, such as Eastern Tumor Collaborative Group performance scores, gene mutation information, and molecular pathology indicators, which may significantly improve the efficacy of existing models. Secondly, our study was retrospective and potential selection bias may have adversely affected the conclusions. Finally, the existence of inconsistent registry staging in the SEER database^[32]^ may have influenced our analysis of prognosis, and our nomograms were only validated internally and further external validation through larger prospective studies is needed.

## 5. Conclusion

In summary, we extracted clinical and pathological factors from the SEER database for patients with AM and found that more than one-third of patients with advanced melanoma died within 3 months. The study also analyzed independent risk factors associated with early death. Based on these factors, we developed and validated nomograms for all-cause early death and cancer-specific early death risk. The good performance of the nomograms suggests that these models can help clinicians identify patients with a high risk of early death and provide them with personalized treatment, thus improving their survival benefit, as well as provide an aid to clinical trial design. Certainly, further studies confirming its practical application in the management of patients with advanced melanoma are of great necessity.

## Author contributions

**Conceptualization:** Bin Liu.

**Data curation:** Bin Liu, Cunli Yin, Xi Yang, Chunyu Wang.

**Investigation:** Cunli Yin, Xi Yang, Yingchun Lu, Chunyu Wang.

**Methodology:** Bin Liu, Siru Li.

**Resources:** Siru Li, Xi Yang, Yingchun Lu.

**Software:** Siru Li, Xi Yang, Yingchun Lu.

**Supervision:** Bin Liu, Cunli Yin, Yingchun Lu.

**Validation:** Siru Li, Chunyu Wang.

**Writing – original draft:** Siru Li.

**Writing – review & editing:** Siru Li.

## References

[1] Ahmed B Muhammad IQ Ghafoor S. Malignant melanoma: skin cancer-diagnosis, prevention, and treatment. Crit Rev Eukaryot Gene Expr. 2020;30:291–7. 32894659 10.1615/CritRevEukaryotGeneExpr.2020028454

[2] Siegel RL Miller KD Jemal A. Cancer statistics, 2019. CA Cancer J Clin. 2019;69:7–34. 30620402 10.3322/caac.21551

[3] Kosary CL Altekruse SF Ruhl J. Clinical and prognostic factors for melanoma of the skin using SEER registries: collaborative stage data collection system, version 1 and version 2. Cancer. 2014;120:3807–14. 25412392 10.1002/cncr.29050

[4] Bedrosian I Faries MB Guerry D. Incidence of sentinel node metastasis in patients with thin primary melanoma (< or = 1 mm) with vertical growth phase. Ann Surg Oncol. 2000;7:262–7. 10819365 10.1007/s10434-000-0262-z

[5] Sundararajan S Thida AM Yadlapati S. Metastatic Melanoma. Treasure Island, FL: StatPearls Publishing; 2022. 29262232

[6] Damsky WE Rosenbaum LE Bosenberg M. Decoding melanoma metastasis. Cancers (Basel). 2010;3:126–63. 24212610 10.3390/cancers3010126 PMC3756353

[7] Gershenwald JE Scolyer RA Hess KR.; for members of the American Joint Committee on Cancer Melanoma Expert Panel and the International Melanoma Database and Discovery Platform. Melanoma staging: evidence-based changes in the American Joint Committee on Cancer eighth edition cancer staging manual. CA Cancer J Clin. 2017;67:472–92. 29028110 10.3322/caac.21409 PMC5978683

[8] Seedor RS Orloff M. Treatment of metastatic melanoma in the elderly. Curr Oncol Rep. 2022;24:825–33. 35316844 10.1007/s11912-022-01257-5

[9] Davis LE Shalin SC Tackett AJ. Current state of melanoma diagnosis and treatment. Cancer Biol Ther. 2019;20:1366–79. 31366280 10.1080/15384047.2019.1640032 PMC6804807

[10] Ye EZ Ye EH Ye RZ. DeepImageTranslator V2: Analysis of Multimodal Medical Images using Semantic Segmentation Maps Generated through Deep Learning. Cold Spring Harbor Laboratory; 2021.

[11] Ying Z Xiongfeng F Lanqing W. A predictive nomogram for early death of metastatic gastric cancer: a retrospective study in the SEER database and China. J Cancer. 2020;11:5527–35. 32742500 10.7150/jca.46563 PMC7391207

[12] Shen H Deng G Chen Q. The incidence, risk factors and predictive nomograms for early death of lung cancer with synchronous brain metastasis: a retrospective study in the SEER database. BMC Cancer. 2021;21:825. 34271858 10.1186/s12885-021-08490-4 PMC8285786

[13] Millet A Martin AR Ronco C. Metastatic melanoma: insights into the evolution of the treatments and future challenges. Med Res Rev. 2017;37:98–148. 27569556 10.1002/med.21404

[14] Ancuceanu R Neagu M. Immune based therapy for melanoma. Indian J Med Res. 2016;143:135–44. 27121512 10.4103/0971-5916.180197 PMC4859123

[15] Jamie G Charlotte A. Update on immunotherapy in melanoma. Surg Oncol Clin N AM. 2015; 24:337–46. 25769716 10.1016/j.soc.2014.12.010

[16] Li W Xiao Y Xu X. A novel nomogram and risk classification system predicting the cancer-specific mortality of patients with initially diagnosed metastatic cutaneous melanoma. Ann Surg Oncol. 2021;28:3490–500. 33191484 10.1245/s10434-020-09341-5

[17] Du Y Li C Mao L. A nomogram incorporating Ki67 to predict survival of acral melanoma. J Cancer Res Clin Oncol. 2023. 10.1007/s00432-023-05127-w PMC10587210 37470854

[18] Cozzolino C Buja A Rugge M. Machine learning to predict overall short-term mortality in cutaneous melanoma. Discov Oncol. 2023;14:13. 36719475 10.1007/s12672-023-00622-5 PMC9889591

[19] Kanaki T Stang A Gutzmer R. Impact of American Joint Committee on Cancer 8th edition classification on staging and survival of patients with melanoma. Eur J Cancer. 2019;119:18–29. 31401470 10.1016/j.ejca.2019.06.011

[20] Swetter SM Tsao H Bichakjian CK. Guidelines of care for the management of primary cutaneous melanoma. J Am Acad Dermatol. 2019;80:208–50. 30392755 10.1016/j.jaad.2018.08.055

[21] Wheatley K Wilson JS Gaunt P. Surgical excision margins in primary cutaneous melanoma: a meta-analysis and Bayesian probability evaluation. Cancer Treat Rev. 2016;42:73–81. 26563920 10.1016/j.ctrv.2015.10.013

[22] Utjés D Malmstedt J Teras J. 2-cm versus 4-cm surgical excision margins for primary cutaneous melanoma thicker than 2 mm: long-term follow-up of a multicentre, randomised trial. Lancet. 2019;394:471–7. 31280965 10.1016/S0140-6736(19)31132-8

[23] Fife KM Colman MH Stevens GN. Determinants of outcome in melanoma patients with cerebral metastases. J Clin Oncol. 2004;22:1293–300. 15051777 10.1200/JCO.2004.08.140

[24] Paek SH Audu PB Sperling MR. Reevaluation of surgery for the treatment of brain metastases: review of 208 patients with single or multiple brain metastases treated at one institution with modern neurosurgical techniques. Neurosurgery. 2005;56:1021–34; discussion 1021. 15854250

[25] Meleti M René LC de Bree R. Head and neck mucosal melanoma: experience with 42 patients, with emphasis on the role of postoperative radiotherapy. Head Neck. 2008;30:1543–51. 18704960 10.1002/hed.20901

[26] Krengli M Masini L Kaanders Johannes HAM. Radiotherapy in the treatment of mucosal melanoma of the upper aerodigestive tract: analysis of 74 cases. A Rare Cancer Network study. Int J Radiat Oncol Biol Phys. 2006;65:751–9. 16647223 10.1016/j.ijrobp.2006.01.016

[27] Hanna TP Chauvin C Miao Q. Clinical outcomes after pulmonary metastasectomy for melanoma: a population-based study. Ann Thorac Surg. 2008;106:1675–81. 10.1016/j.athoracsur.2018.06.078 30171851

[28] Bello DM Panageas KS Hollmann T. Survival outcomes after metastasectomy in melanoma patients categorized by response to checkpoint blockade. Ann Surg Oncol. 2020;27:1180–8. 31848819 10.1245/s10434-019-08099-9 PMC7112166

[29] Klemen ND Shindorf ML Sherry RM. Role of surgery in combination with immunotherapy. Surg Oncol Clin N Am. 2019;28:481–7. 31079801 10.1016/j.soc.2019.02.011

[30] Ascierto PA Long GV Robert C. Survival outcomes in patients with previously Untreated BRAF wild-type advanced melanoma treated with nivolumab therapy: three-year follow-up of a randomized phase 3 Trial. JAMA Oncol 2019; 5(2):187–194 30422243 10.1001/jamaoncol.2018.4514 PMC6439558

[31] Sperduto PW Kased N Roberge D. Summary report on the graded prognostic assessment: an accurate and facile diagnosis-specific tool to estimate survival for patients with brain metastases. J Clin Oncol. 2012;30:419–25. 22203767 10.1200/JCO.2011.38.0527 PMC3269967

[32] Cullison CR Zheng DX Levoska MA. Inconsistencies in cutaneous melanoma staging Within SEER Registries. JAMA Dermatol 2018;157:727–9. 10.1001/jamadermatol.2021.1098 PMC8100907 33950199

